# Strain–Microstructure–Optoelectronic Inter-Relationship toward Engineering Mechano-Optoelectronic Conjugated Polymer Thin Films

**DOI:** 10.3390/polym13060935

**Published:** 2021-03-18

**Authors:** Youngmin Lee, Alfred Mongare, Aaron Plant, Donghyeon Ryu

**Affiliations:** 1 Department of Chemical Engineering, New Mexico Tech, Socorro, NM 87801, USA; aaron.plant@student.nmt.edu; 2 Department of Mechanical Engineering, New Mexico Tech, Socorro, NM 87801, USA; alfred.mongare@student.nmt.edu

**Keywords:** mechano-optoelectronics, conjugated polymers, microstructures, poly(3-hexylthiophene), strain sensors

## Abstract

Mechano-optoelectronic (MO) behavior indicates changes in optoelectronic properties in response to the applied mechanical deformation. The MO behavior can be employed to monitor the mechanical deformation of a targeted system by tracing its optoelectronic properties. Poly(3-hexylthiophene) and phenyl-C_61_-butyric acid methyl ester (P3HT/PCBM) blend thin films exhibited changes in direct current under tensile strain. Although optoelectronic properties and photovoltaic performance of P3HT/PCBM blends have been studied extensively and intensively, research required for MO properties has a fundamental difference from previous research mostly for solar cells. In research for MO systems, a greater extent of changes in optoelectronic properties under mechanical deformation is favorable. Herein, previous research for optoelectronic properties and mechanical properties of conjugated polymers will be reviewed from a perspective on MO properties. The microstructure of a conjugated polymer thin film plays a pivotal role in its optoelectronic properties and mechanical properties. Key parameters involved in the microstructure of conjugated polymer thin films will be addressed. A scalable process is required to broaden applications of MO systems. Potential challenges in the fabrication of MO conjugated polymer thin films will be discussed. Finally, this review is envisioned to provide insight into the design and manufacturing of MO conjugated polymer thin films.

## 1. Introduction

Conjugated polymers have broadened their engineering application thanks to the unique multifunctionality of exhibiting optoelectronic properties and mechanical flexibility by creating novel electronics that traditional inorganic semiconductors were not able to make available [1]. To name a few, organic photovoltaics (OPVs), organic light-emitting diodes (OLEDs), and organic thin-film transistors (OTFTs) could not have been invented without the conjugated polymers [2,3,4,5,6,7,8,9]. In future, it is envisioned that the flexible electronics made with conjugated polymers will be prevalent to be instrumented to more objects and biological systems due to lightweight and flexibility features [10,11,12].

OPVs have shown promising potential as an energy harvesting component of wearables, smart glass, building façade, and deployable space structures, among others [13]. The OPVs’ radiant-electrical energy conversion mechanism is fundamentally identical to the mechanism of the inorganic PVs, which creates an exciton (i.e., a couple of hole and electron) at a p-n junction upon impinging of photons to result in dissociation of the hole and electron, which are consequently pushed toward one and the other electrodes under potential gradient to finally create a direct current [14]. The main difference between the inorganic PVs and OPVs is, in general, how the p-n junctions are created. Unlike the inorganic PVs that have layered up p- and n-semiconductors to form a plane junction, OPVs have dissociation spots in p-n bulk heterojunction (BHJ) structures [15,16]. The BHJ is made where conjugated polymers as a p-type semiconductor and an n-type semiconductor create interfaces. The 3D BHJ structures is mainly determined by formation of microstructures of the conjugated polymers molecular scale. Therefore, the majority of studies about OPVs have focused on how to optimize the BHJ structures by ordering the microstructures of the conjugated polymers in favor to maximize power conversion efficiency (PCE) [16,17].

A strong relationship between microstructures and optoelectronic properties of the conjugated polymers needs to be taken into account when designing flexible electronics as those can potentially undergo mechanical deformation that results in a change in microstructures. While there are pioneering studies about how microstructures of conjugated polymers affect optoelectronic properties and mechanical properties of the conjugated polymer BHJ structures, there is much less attention to the knowledge gap about how optoelectronic properties vary with the degree of deformation of the conjugated polymer BHJ thin films, which is induced by mechanical strains applied onto the thin films. More attention in the materials science community is on enlightening how the mechanical strain applied onto the BHJ thin films affects the microstructures of the conjugated polymers and thus hole and electron dissociations and transfers at the molecular scale. The efforts in the solar cell community naturally are towards maintaining optoelectronics when the thin films are subjected to mechanical deformations [18,19,20,21].

**Figure 1:**
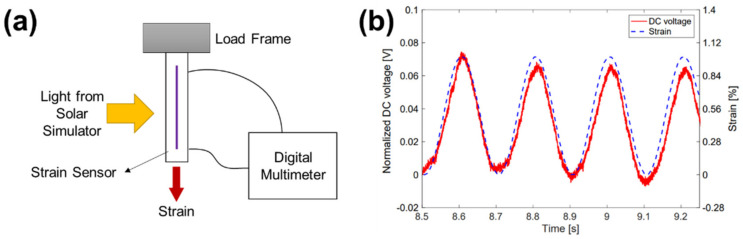
(**a**) Illustration of the experimental setup to observe mechano-optoelectronic behavior of a poly(3-hexylthiophene) and phenyl-C_61_-butyric acid methyl ester (P3HT/PCBM) blend thin film that exerts tensile stress while monitoring the photocurrent. (**b**) DC voltage response of a P3HT/PCBM thin film normalized by the magnitude at 0% strain is overlaid to a pattern of the applied tensile stress cycles. Reprinted from [25].

From the engineer’s perspective, varying optoelectronic properties due to change in microstructures of the thin films subjected to mechanical deformation are considered a unique material property of the conjugated polymers. Mechano-optoelectronic (MO) behavior indicates varying optoelectronic properties of the conjugated polymer thin films subjected to the applied mechanical deformation. Using the MO behavior, the mechanical deformation of a targeted system can be monitored by tracking changes of its optoelectronic properties. Pioneering works by Ryu and Loh [22,23,24,25] presented possibilities of conjugated polymer thin films as a mechano-optoelectronic system. Poly(3-hexylthiophene) and phenyl-C_61_-butyric acid methyl ester (P3HT/PCBM) blend thin films exhibited changes in photocurrent under tensile strain. P3HT/PCBM blend thin films were prepared on flexible substrates, such as an indium tin oxide (ITO) sputtered polyethylene terephthalate (PET) substrate [22,23,24] and a polydimethylsiloxane (PDMS) substrate coated with a PEDOT:PSS electrode [25]. When the tensile strain was applied to the thin film device, the DC voltage, which was generated from the device under light, changed along with cycles of tensile stress as shown in Figure 1. This response of photocurrent to the applied tensile strain can be utilized as a strain sensing signal in structural health monitoring, biomedical devices, and wearable devices, among others.

P3HT/PCBM blends are one of the most representative solar cell systems that have been studied extensively and intensively with respect to optoelectronic properties and photovoltaic performance [7,8,9,16,26]. Although new systems that exhibit superior photovoltaic performance have been developed, for example, all-polymer systems substituting PCBM as electron acceptor polymers and systems with new donor polymers instead of P3HT [5,21,27,28,29], P3HT/PCBM blend thin films needs to be revisited because of their MO properties. In this review article, previous research for optoelectronic properties and mechanical properties of P3HT/PCBM thin films will be reviewed in perspective of MO properties. The microstructure of a conjugated polymer thin film plays a pivotal role in its optoelectronic properties and mechanical properties. Key parameters involved in the microstructure of P3HT/PCBM blend thin films will be addressed in terms of regioregularity, crystallinity, orientation, and blend morphology. Determination of the parameters and their correlation to optoelectronic and mechanical properties will be described for the design of MO systems. Next, manufacturing of MO conjugated polymer thin films will be discussed. Scalable processes need to be adopted to broaden applications of MO systems in various fields. Due to similarity in device architecture, the fabrication of MO devices is anticipated to face similar challenges that the fabrication of large-scale solar cells has. Approaches in previous research will be discussed to address these challenges in scalable processes, such as inkjet printing, spray coating, and roll-to-roll printing.

## 2. Design of Conjugated Polymer MO Systems

### 2.1. Regioregularity

**Figure 2:**
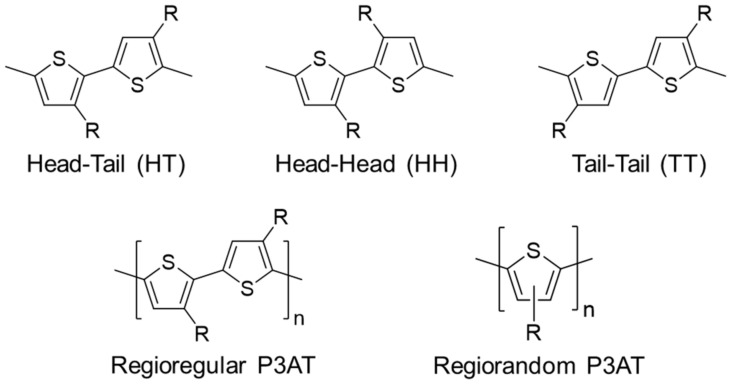
Chemical structures of three regioisomers in 3-alkyl thiophene dimers (**top**) and chemical structures of regioregular and regiorandom poly(3-alkyl thiophene)s (**bottom**).

Due to its asymmetric chemical structure, dimers of 3-alkyl thiophene have three different regioisomers: Head–Tail (HT), Head–Head (HH), and Tail–Tail (TT). Chemical structures of the three regioisomers and poly(3-alkyl thiophene) (P3AT) are presented in Figure 2. Among the three, the HT isomer is regioregular, and the HH and TT are regioirregular. A polymer chain comprised of the sole HT linkage is called regioregular P3AT. A polymer chain comprised of a mixture of the HT, HH, and TT linkages are called regioirregular P3AT. If it has an arbitrary mixture of the regioregular and regioirregular linkages, a polymer chain is called regiorandom P3AT. Regioregular P3AT typically exhibits superior optoelectronic properties than those of regioirregular P3AT due to more planar fully conjugated polymer backbones. Regioregularity of P3AT can be determined by ^1^H NMR or MALDI-ToF [30,31,32,33,34].

**Figure 3:**
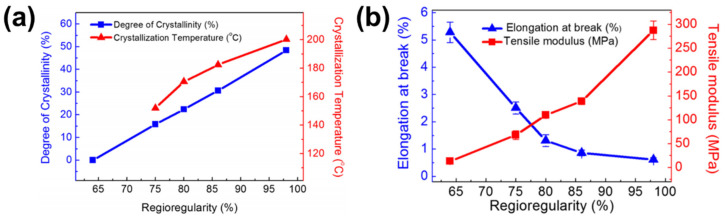
(**a**) Degree of crystallinity and crystallization temperature of P3HT as a function of regioregularity and (**b**) elongation at break and tensile modulus of P3HT thin films with different regioregularity. Reprinted with permission from [38]. Copyright 2016 American Chemical Society.

The difference in the configuration of monomers affects density [35], crystallinity [36], glass transition temperature [37], mechanical properties [38], and optoelectronic properties, for example, UV-V is absorption, energy levels, and charge transport [38,39,40]. Lee and Dadmun [35] reported density of regioregular and regiorandom P3HT samples measured by gas pycnometry: 1.105–1.108 g/cm^3^ for regioregular P3HT depending on its crystallinity and 1.111 g/cm^3^ for regiorandom P3HT. Using the correlation between density and crystallinity, the density of the crystalline region (i.e., 100% crystallinity) and amorphous (0% crystallinity) region of regioregular P3HT was estimated by extrapolation of the experimental data. The density of the amorphous region in regioregular P3HT was 1.094 g/cm^3^ and that of the crystalline region was 1.12–1.14 g/cm^3^. Interestingly, the density of regiorandom P3HT differed from the density of the amorphous phase of regioregular P3HT. It was inferred that chain packing and thermodynamic behavior of amorphous phase of regioregular P3HT should be different from those of regiorandom P3HT [35]. Regioregular P3HTs have planar backbones that allow regular chain packing and thus higher crystallinity. In the presence of a head–head regioisomer, however, the HH linkage causes a kink in the chain. The kink acts as a defect site that hinders regular chain packing. Although it can be incorporated in the crystal, the kink causes a considerable free energy penalty, as a result, it drops degree of crystallinity of P3HT. For this reason, as shown in Figure 3a, the degree of crystallinity of P3HT decreased from 48% to 16% as regioregularity decreased from 98% to 75% and P3HT having regioregularity of 64% or less showed 0% crystallinity, i.e., regiorandom P3HT was amorphous [36,38].

Since chain packing changes with regioregularity, the regioregularity affects chain motion as a function of temperature or gives a major impact on glass transition. Glass transition brings a significant change in the viscoelastic behavior of a polymer [41]. By definition, polymer chains cannot move below the glass transition temperature. As a result, the glass transition temperature of conjugated polymers will be a lower limit temperature to operate a MO system. The glass transition temperature of P3HT samples has been determined by differential scanning calorimetry (DSC) [42,43]. Glass transition in P3HT thin films can be determined by monitoring changes in the polarization state (e.g., amplitude ratio and phase shift) or film thickness as a function of temperature using in-situ ellipsometry [44,45]. However, reported values of the glass transition temperature of P3HT vary substantially [43,45,46,47,48,49]. Xie et al. [37] revealed that P3HT had two glass transitions using oscillatory shear rheometry: one between −11 and 15 °C from vitrification of the polymer backbone and one between −94 and −101 °C from side-chain motion. The glass transition temperature from the backbone elevated as the molecular weight increased, while the glass transition temperature from the side-chain was independent of the molecular weight. This trend for the glass transition temperature as a function of molecular weights was different depending on the regioregularity. Regioregular P3HT gave consistently higher glass transition temperature associated with the backbone than glass transition of regiorandom P3HT. The backbone glass transition temperature extrapolated to the infinite molecular weight was 22 °C for regioregular P3HT and 6 °C for regiorandom P3HT. Storage modulus above the glass transition temperature was orders of magnitude higher in regioregular P3HT than in regiorandom P3HT [37].

Mechanical properties of conjugated polymers vary depending on regioregularity. Kim et al. [38] prepared a series of P3HT samples with the incorporation of a dimer having the head–head linkage in polymerization to control regioregularity. As regioregularity decreased from 98% to 64%, tensile moduli of P3HT thin films decreased from 290 to 13 Mpa, as seen in Figure 3b. As opposed to the trend for tensile modulus, elongation at break of P3HT thin films increased from 0.8% to 5.4% under the decrease in regioregularity from 98% to 64%. A P3HT thin film of higher regioregularity has more portion of ordered regions that has intermolecular interaction through secondary bonds, such as van der Waals and π-π interaction. Under tensile strain, those secondary bonds inhibit deformation, which in turn give stronger tensile modulus, but smaller elongation at break. In contrast, a P3HT thin film of lower regioregularity has less ordered regions and more soft and ductile amorphous regions, which gives smaller tensile modulus, but greater elongation at break [38].

The optoelectronic properties of conjugated polymers are also affected by regioregularity. UV-V is absorption spectra of regioregular P3HT thin films exhibit the characteristic feature around 600 nm involved in ordered structures, whereas there are no signs of ordering from a regiorandom P3HT thin film [39,40]. This difference in UV-V is absorption causes different optical band gaps depending on regioregularity. The optical band gap in thin films was estimated as 1.9 eV for regioregular P3HT and 2.2 eV for regiorandom P3HT. The highest occupied molecular orbital (HOMO) energy levels of P3HT thin films were estimated by UV photoelectron spectroscopy (UPS). The HOMO level of regioregular P3HT was −4.9 eV and that of regiorandom P3HT was −4.6 eV. This 0.3 eV higher HOMO level from regioregular P3HT was attributed to a highly ordered structure where extraction of the electron could arise readily [50]. For comparison, HOMO and the lowest unoccupied molecular orbital (LUMO) energy levels for PCBM were estimated as −5.8 and −4.0 eV, respectively [39]. The charge carrier mobility of the P3HT thin films followed the same trend as that of UV-V is spectra. A regiorandom P3HT thin film exhibited a slower and more dispersive charge transport than the charge transport in regioregular P3HT thin films, which had a higher fraction of the ordered P3HT structure. As regioregularity decreased 98% to 75%, charge carrier mobility drops from 10^−1^ to 10^−4^ cm^2^/V∙s. A drastic drop in charge carrier mobility appeared by further decreasing regioregularity. A P3HT thin film with regioregularity of 64% exhibited 10^−8^ cm^2^/V∙s [38]. When P3HT samples with different regioregularity were employed for solar cell devices coupled with PCBM, P3HT with a relatively lower regioregularity (94%) showed lower hole mobility via the P3HT phase than electron mobility via the PCBM phase, i.e., an unbalanced charge transport. A device made using P3HT with a higher regioregularity (98%) exhibited balanced hole mobility to electron mobility. Nevertheless, solar cell devices from both P3HT samples gave similar PCE of 2.5% [40].

As mentioned above, regioregularity affects crystallinity, glass transition, UV-vis absorption, and charge transport, which are all involved in MO properties. Considering the result that solar cell devices using high and medium regioregularity P3HT coupled with PCBM give comparable PCE [40], regioregularity needs to be studied and optimized for MO performance. Potentially P3HT with a medium range of regioregularity can be a promising material to improve mechanical properties and sensitivity under mechanical deformation without sacrificing device performance. From this point forward, all references to P3HT refer to regioregular P3HT unless specified as regiorandom P3HT.

### 2.2. Crystallinity

**Figure 4:**
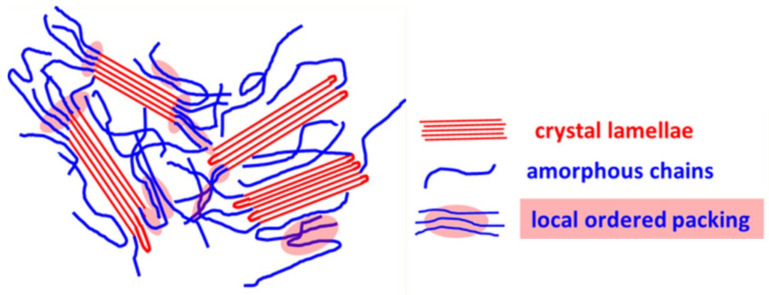
Illustration of the microstructure of semicrystalline conjugated polymers. There coexist ordered region, amorphous region, and an intermediate region in-between. Reprinted with permission from [51]. Copyright 2016 American Chemical Society.

Conjugated polymers are known as “semicrystalline.” The microstructure of a conjugated polymer thin film is composed of a crystalline region (highly ordered), an amorphous region (disordered), and an intermediate region (locally ordered) in-between, as illustrated in Figure 4. The degree of crystallinity of conjugated polymers can be estimated by density, DSC, X-ray diffraction, and solid-state NMR [35,51,52,53]. The amorphous region and crystalline region of conjugated polymers have different densities of chain packing and thus different mass densities. Using the densities of completely crystalline P3HT and completely amorphous P3HT, proportional distribution for a density of unknown sample yielded mass fraction or volume fraction of crystalline portion, which is the degree of crystallinity. Densities of the completely crystalline and completely amorphous P3HTs were estimated by extrapolation based on densities of P3HTs of known crystallinity. In the case of the completely crystalline P3HT, a density could also be calculated from a crystal lattice [35,51]. The degree of crystallinity can be characterized by analysis of the enthalpy of fusion of the crystalline portion. The most direct approach to estimate the absolute degree of crystallinity is a comparison to the enthalpy of fusion from the completely crystalline P3HT. However, it has been difficult to obtain a trustworthy value for the completely crystalline P3HT. Alternatively, Snyder et al. [52] studied the correlation between the enthalpy of fusion and the size of crystallites using P3HT samples of known crystallinity. The enthalpy of fusion of a crystalline portion of a P3HT sample decreased with the smaller size of crystallites due to effects by the finite size of crystallites [52]. The degree of crystallinity can also be estimated by wide-angle X-ray diffraction (WAXD) and solid-state NMR. Deconvolution of a WAXD or NMR profile into the contribution of amorphous phase and crystalline phase yielded the degree of crystallinity of P3HT. A profile of a regiorandom P3HT was employed as a reference for the completely amorphous (i.e., 0% crystallinity) state. The degree of crystallinity of P3HT varied on the measurement technique. Crystallinity estimated by solid-state NMR was around 10% greater than that by WAXD. It was attributed to local short-range ordering at the interface between crystalline and amorphous regions (red area in Figure 4). The local ordering did not create diffraction signals detectable by WAXD, while NMR could detect interaction from the local ordering [51,52,53].

Crystallinity depends on the molecular weight [51,52,54,55], regioregularity [36,38], and processing conditions [26,56,57,58,59]. The crystallinity of P3HT decreases from 60% to 46% as molecular weight increases from 8 to 30 kg/mol measured by WAXD and DSC analysis [51,52]. Low-molecular-weight P3HT near or shorter than the persistence length, 3 nm [60,61], has rod-like nature, which is favorable for the ordered structure. In contrast, high-molecular-weight P3HT longer than the persistence length is flexible. It has a more disordered phase by bending or folding of the backbone leading to less crystallinity. For processing solvent, high boiling point solvent allows sufficient time to develop microstructures of conjugated polymers during the spin coating process, which results in a higher degree of crystallinity [56]; in a similar manner, thermal annealing and solvent vapor annealing allow local mobility of conjugated polymer chains, which facilitates the highly ordered microstructure [57,58].

The degree of crystallinity plays a key role in optoelectronic properties, such as charge carrier mobility [54,55,56,62], and as a result, the performance of solar cell devices [26,39,59]. However, the charge carrier mobility of conjugated polymers is not simply proportional to the degree of crystallinity. For example, a thin film with low-molecular-weight P3HT creates highly ordered structures associated with characteristic nanofibril structure, but it exhibits lower charge carrier mobility than a thin film with high molecular weight [54,55]. Different processing solvents make charge carrier mobility of conjugated polymer thin films three times higher than another thin film despite having the same degree of crystallinity [56]. These two examples imply that the charge carrier mobility is not only dependent on crystallinity but also depends on another factor, i.e., the interconnectivity of ordered regions. The charge carrier mobility of P3HT thin films was investigated as a function of molecular weight. A P3HT thin film with low molecular weight (<4 kg/mol) was comprised of packed nanofibrils, whereas one with high molecular weight (>30 kg/mol) has rather isotropic morphology with less clear nanofibrils. Despite the highly ordered microstructure, the film with low molecular weight showed two orders lower charge carrier mobility than that of high molecular weight. The result was attributed to a poor connection between ordered regions in low-molecular-weight P3HT. Although it gave excellent charge transport within an ordered region, the P3HT film with low molecular weight had the energy barrier at the boundary of an ordered region. On the other hand, the P3HT film with high molecular weight had no such energy barrier and provided more pathways across the entire thin film [54,55]. These pathways can form by tie chains that connect adjacent ordered reasons. Tie chains appear when the molecular weight of a conjugated polymer is sufficiently large, thus when the end-to-end distance is greater than the distance between adjacent ordered regions. These tie chains establish intercrystallite connectivity to bypass the energy barriers in amorphous regions, which permits higher charge carrier mobility [63]. Similar to charge carrier mobility as above, a universal correlation may not exist between crystallinity and MO properties. Other parameters for microstructure need to be considered simultaneously.

### 2.3. Orientation

**Figure 5:**
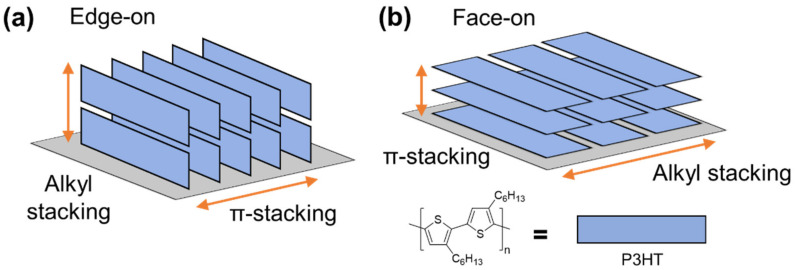
Illustration for orientation of P3HT on a substrate: (**a**) edge-on and (**b**) face-on.

Conjugated polymers have anisotropic crystal structures. If chemical structures of conjugated polymers are simplified to a planar backbone that has alkyl side chains extending from the edges, three directions in a crystal structure indicate one through both alkyl side chains and planar backbones (or alkyl stacking), one through planar backbones (or π-stacking), and one along the backbone. In conjugated polymer thin films, there are two different orientations with respect to the substrate: “face-on”, which aims at the planar backbone parallel to the surface and “edge-on”, which aims at the alkyl side chains perpendicular to the surface. P3HT crystal structure and orientation on the substrate are illustrated in Figure 5. These different orientations have significant impacts on optoelectronic properties, such as charge carrier mobility and UV-Vis absorption, and consequently, device performance [64,65,66,67,68,69,70]. Charge transport along the backbone and through π-stacking occurs by intrachain and interchain transport mechanisms, respectively. While de-localized electrons in conjugated π-orbitals give the highest mobility, the intrachain transport mechanism is limited by the dimension of a P3HT chain. For this reason, interchain transport mechanism by charge hopping through π-π interactions within a crystallite is the main pathway throughout a thin film. In contrast to these two pathways, charge transport declines significantly through alkyl stacking [71]. Depending on the orientation of P3HT chains, charge transport in a thin film occurs through different pathways. In thin-film transistors (TFTs), charge carrier mobility increased 100-fold in a P3HT thin film with edge-on orientation (i.e., charge transport through π-stacking) than a thin film with face-on orientation (i.e., charge transport through alkyl stacking) [64].

Due to the anisotropy in the crystal structure, a certain type of orientation is more favorable than the other, depending on applications. Substrate modification is used to manipulate the orientation of P3HT chains in a thin film. Self-assembled monolayers (SAMs) use small molecules capable of anchoring the surface of a substrate, such as hexamethyldisilazane (HMDS) and octadecyl trichlorosilane (OTS). Depending on their chemical structure and number density on the surface, SAMs control the surface energy of a substrate from hydrophilic to hydrophobic that align polymer chains [64,72]. For the orientation control by SAMs, the thickness of a film needs to be considered as vertical confinement. The bottom surface of a film has higher alignment crystallites directed by SAMs, whereas the bulk region has the random orientation of crystallites [73]. Therefore, a thicker film may require further approaches to control the overall orientation of P3HT. Slow evaporation of a processing solvent induces improved ordering over the formation of microstructure. Spin coating under a high vapor pressure of a processing solvent or drop-casting allows slower evaporation than spin coating in the atmosphere (i.e., zero vapor pressure), which in turn induces highly ordered edge-on microstructure in a P3HT thin film [65]. Solubility control by means of selection of a processing solvent and temperature of a substrate can improve the ordering of microstructure in a P3HT thin film [70]. Epitaxy uses crystalline substrates to obtain directional crystallization of conjugated polymers. For the orientation of P3HT, a thin film is prepared with a mixture of P3HT and 1,3,5-trichlorobenzene (TCB), and then heated at 85 °C and cooled down for melting and crystallization of TCB. On the surface of the TCB layer, P3HT chains are subsequently rearranged along the direction of crystalline TCB [74,75,76]. External forces are also used to align P3HT chains in a thin film. The polymer backbones of P3HT align in the direction of mechanical rubbing [77,78]. Another approach uses uniaxial elongation, and then the aligned P3HT chains can be transferred onto another substrate by PDMS imprinting [67,68,69,79,80]. There is also an approach using surface tension. Conjugated polymer chains are floated onto water surface as a monolayer and then confined to obtain a thin film with aligned polymer chains [81,82,83]. These approaches using external forces provide in-plane alignment that is commonly not attainable through the aforementioned approaches. Therefore, a combination of these approaches will be an effective strategy to obtain a higher level of ordering in the microstructure of a P3HT thin film that controls both in-plane alignment and orientation with respect to the substrate.

**Figure 6:**
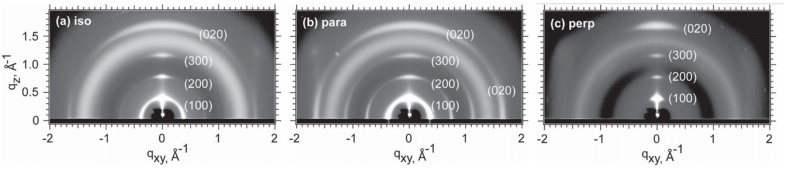
Grazing incidence X-ray diffraction images of P3HT thin films (**a**) before tensile strain applied and under 100% strain (**b**) parallel to incident X-ray beam and (**c**) perpendicular to the incident X-ray beam. Reprinted with permission from [84]. Copyright 2015 John Wiley and Sons.

Since conjugated polymer films have random orientation, the impact of the orientation is not typically observed. O’Connor et al. [67,68,84] have explored how microstructural parameters of P3HT affect the anisotropic electrical properties. P3HT thin films were uniaxially strained so that the polymer backbone became aligned along the direction of strain. In UV-Vis absorption spectroscopy using a polarized beam, the absorption profile under polarized light parallel to the strain direction gave a red-shift as a function of strain, while it gave blue-shift under polarized light perpendicular to the strain direction. The anisotropy increased with the extent of strain. This indicated that more fraction of P3HT backbones were aligned to the direction of strain. In grazing incidence X-ray scattering, as shown in Figure 6, the orientation of P3HT chains became more face-on dominant as a function of strain, while the spacing for alkyl stacking and π-stacking was independent of strain. Therefore, P3HT crystallites tended to rotate toward the strain direction as well as toward face-on orientation under strain [67,84]. These changes in microstructure induced anisotropic charge carrier mobility. The charge carrier mobility of a P3HT thin film parallel to the strain direction increased 4 times at 140% strain compared to that at 0% strain. Charge carrier mobility along with perpendicular to the strain direction declined around half; as a result, an anisotropy ratio in charge carrier mobility rose up to 9 at 140% strain of a P3HT thin film. Surprisingly, thermal annealing reduced charge carrier mobility along with both parallel and perpendicular directions. Thermal annealing did not bring significant changes in the population of ordered chains or a type of orientation. Instead, the reduction of charge carrier mobility after thermal annealing was attributed to local rearrangement of P3HT chains that led to the removal of tie chains between ordered regions [68,85]. This is another example representing the importance of interconnectivity of ordered regions for charge transport.

Aligning a thin film of P3HT/PCBM blend using a uniaxial strain can also permit a significant increase in photovoltaic performance [69,84]. At 50% strain, the short circuit current (*J*_SC_) increased with similar fill factor (*FF*) and open-circuit voltage (*V_OC_*) under parallelly polarized light to the strain direction. The increment is attributed to an increase in absorptivity. This allowed for a higher external quantum efficiency (EQE), particularly around 600 nm involved in light absorption by ordered P3HT regions. In contrast, under perpendicularly polarized light, the same sample at 50% strain gave drastic drops in *J*_SC_ and EQE around 600 nm. It implied perpendicularly polarized light could not contribute to absorption or the following process for charge generation in a solar cell device. These parameters decreased at further strain up to 100% under both directions of polarization due to thinning effect of the overall film thickness. PCE of the uniaxially elongated device coincides with *J*_SC_ and EQE as a function of strain, i.e., increase at 50% strain, but decrease at further strain under parallelly polarized light to the strain direction. Under perpendicularly polarized light, PCE continued to decrease up to 100% strain. Different from device parameters, the ratios of absorbance and *J*_SC_ depending on the direction polarization continued to increase up to 100% strain; thus, a solar cell device became more sensitive to the polarization of incident light as the strain increased [69].

**Figure 7:**
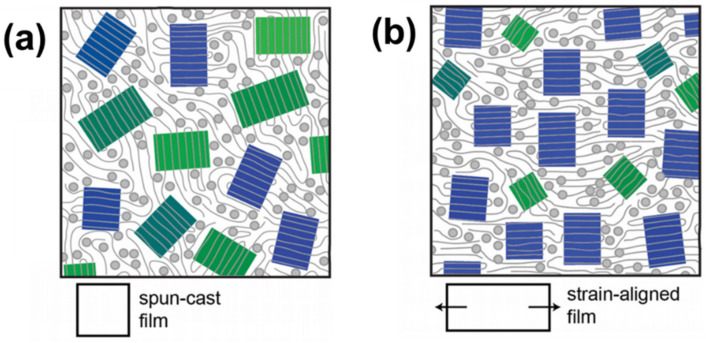
Illustration for orientation of P3HT crystallites in P3HT/PCBM blends thin films. Blue rectangles represent P3HT crystallites aligned to a direction of strain. Green rectangles represent those not aligned. (**a**) Initially, it has a random orientation, i.e., a mixture of blue and green rectangles. (**b**) Under tensile strain, more crystallites are aligned to the strain direction. Reprinted with permission from [84]. Copyright 2015 John Wiley and Sons.

A thin film of P3HT/PCBM blend has random orientation initially, as illustrated in Figure 7a. Under tensile strain, the thin film is anticipated to have more population of the P3HT backbone parallel to the strain direction, which is illustrated as blue rectangles in Figure 7b. For the out-of-plane direction, a thin film of P3HT/PCBM has a more face-on dominant orientation under tensile strain. MO properties should be intimately correlated to these changes in orientation. For this reason, manipulating the initial microstructure can offer opportunities to improve MO sensing. For example, a greater extent of changes in orientation under elongation can be expected from initially edge-on dominant orientation than from initially face-on orientation. If the initial microstructure has an in-plane alignment to a specific direction, greater change in the microstructure can be detected when the strain direction is perpendicular to the initial alignment, compared to the case when the strain direction is parallel to the initial alignment. The size of crystallites determines the agility of rotation under tensile strain; therefore, it is also an important factor to be optimized. Lastly, the interconnectivity of ordered regions needs to be studied for MO behavior. In addition, polarized light parallel to the strain direction is found to be absorbed more than that perpendicular to the strain direction. Using Polarized incident light is expected to provide enhanced sensitivity and directional sensitivity of MO detection.

### 2.4. Blend Morphology

**Figure 8:**
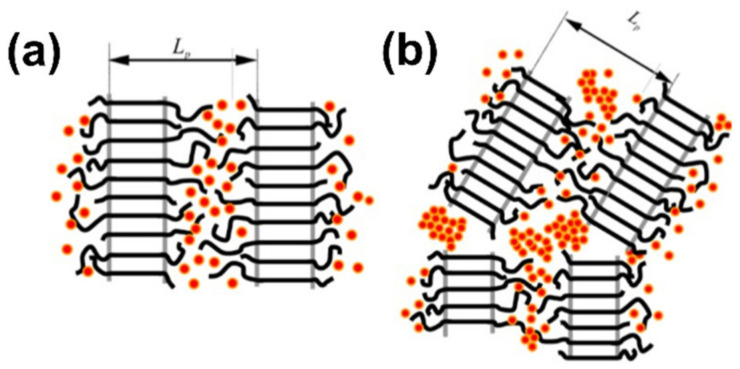
Illustration of the microstructure of P3HT/PCBM blends. (**a**) Below a threshold fraction of PCBM, PCBM is mixed into the amorphous region of P3HT. (**b**) Above the threshold, PCBM segregates out of the amorphous P3HT region and forms aggregates. Reprinted with permission from [87]. Copyright 2013 American Chemical Society.

In a P3HT/PCBM blend, two components are partially miscible. PCBM is mixed into the amorphous P3HT region, while the crystalline P3HT region remains pure. Below a threshold fraction of PCBM, the microstructure of P3HT/PCBM blends consists of crystalline P3HT and a mixture of amorphous P3HT/PCBM, as seen in Figure 8a. Above the threshold, PCBM segregates out of the amorphous P3HT region and forms aggregates, as illustrated in Figure 8b [86,87]. The microstructure of P3HT/PCBM blends is of great importance for the overall process of power generation in solar cell devices, including charge separation and charge transport. The microstructure of P3HT/PCBM blends has been controlled by processing conditions, such as composition [86,88,89], processing solvent [26,90], processing additives [91,92,93,94], and post-treatment, such as thermal annealing and solvent vapor annealing [57,58,89,95,96,97,98,99,100].

**Figure 9:**
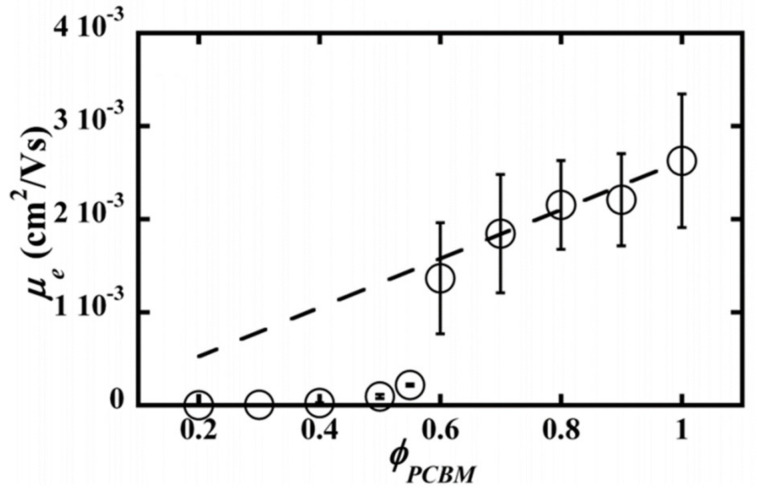
Electron mobility of P3HT/PCBM field-effect transistors at various PCBM volume fractions. Reprinted with permission from [86]. Copyright 2012 American Physical Society.

The composition of a P3HT/PCBM blend has a profound effect on its microstructure, which in turn leads to an effect on charge transport and the overall performance of a device. Vakhshouri et al. [86] examined the microstructure and electron mobility of P3HT/PCBM blends as a function of the PCBM fraction, as shown in Figure 9. When the volume fractions of PCBM decreased from 1.0 to 0.2 in P3HT/PCBM blends, the electron mobility declined gradually because electron transport occurred through the PCBM phase. At the PCBM volume fraction of 0.6, however, the electron mobility showed a sudden drop with orders of magnitude difference. This result was attributed to the phase transition from the immiscible state to the miscible state in P3HT/PCBM blends. Energy-filtered TEM imagery showed that P3HT/PCBM blends with high PCBM volume fractions (>0.6) had segregated PCBM domains that provided percolating pathways for enhanced electron mobility. In contrast, no such phase separation was visible in blends with low PCBM volume fraction (<0.6) [86]. For the overall device performance, too high a fraction of PCBM would cause an adverse effect on hole mobility through the P3HT phase. Therefore, partial miscibility in a P3HT/PCBM blend is critical for efficient photovoltaic performance.

Processing solvents give significant impacts on the microstructure of a P3HT/PCBM blend. Even though a processing solvent is a good solvent for the entire blend, its solubility for each component may differ. Depending on its solubility for P3HT or PCBM, a processing solvent can be either selective or non-selective. Thin films of P3HT/PCBM blends were prepared from chlorobenzene, toluene, and mixtures of the two solvents as processing solvents. Both chlorobenzene and toluene were known to be good solvents for P3HT/PCBM blends. Toluene, however, was rather selective to P3HT, whereas chlorobenzene was non-selective. Due to its selectivity to P3HT, thin films made with toluene had more obvious PCBM aggregation than those made with non-selective chlorobenzene [90]. As stated in the previous paragraph, PCBM aggregation plays a key role in creating the percolating pathways for electron transport. Nonetheless, bulky PCBM aggregation hinders efficient hole transport and declines the interface area between P3HT and PCBM for charge separation.

After a P3HT/PCBM thin film is prepared, the initial microstructure can be tuned by post-treatment using thermal annealing and solvent vapor annealing. Both thermal annealing and solvent vapor annealing accord molecular mobility in the thin film in order to improve the initial microstructure. An essential point in the annealing process is that the molecular mobility induced by the annealing process should be controlled less than a level leading to an abrupt change in microstructure or dewetting of the entire film. For this reason, the thermal annealing temperature is generally determined above a glass transition temperature of PCBM (around 113 °C) [95] and below a melting temperature of P3HT (200 to 230 °C depending on molecular weight) [37,39,89]. In solvent vapor annealing, mild vapor pressure is used to allow solvation of P3HT and PCBM molecules by solvent vapor permeating a thin film, yet to prevent dissolving the molecules [58,98]. These post-treatments induce the self-assembly of molecules toward a more thermodynamically stable state, which increases the ordered region of P3HT and aligns the orientation of P3HT crystallites. As a result, the overall performance of a solar cell device can be enhanced by the post-treatments [95,96,98,99,100].

Processing additives in P3HT/PCBM solutions are also a very effective approach to control the microstructure of thin films. Relevant research has shown that the addition of a small amount of processing additive into the solution before casting can improve the microstructure of a P3HT/PCBM active layer and consequently increase the overall performance [91,92,93]. Although the underlying mechanism has not yet been understood completely, control of microstructure by additives is explained in two different ways: a high boiling point and selective solubility. Additives have a higher boiling point than that of the host solvent. The additives retard the solidification of P3HT chains in thin-film casting processes, which allows self-assembly of P3HT chains to develop a highly ordered structure. The additives have selective solubility to PCBM that shifts solubility of the solvent mixture toward a PCBM favored environment. This change substantially mitigates the formation of PCBM aggregation in thin-film casting processes. A widely used additive 1,8-diiodooctane (DIO) was examined in P3HT/PCBM thin films and compared to those without the addition of DIO. In UV-vis absorption spectra, the overall absorbance of a thin film prepared with the addition of DIO was stronger than that prepared without DIO. Especially absorption around 600 nm was significantly enhanced, which implied the formation of a higher ordering structure in the P3HT phase. Consistent with the enhanced absorbance, a P3HT/PCBM thin film with DIO exhibited enhanced EQE around 600 nm; as a result, the PCE from a solar cell device with the addition of DIO increased 30% in comparison to a reference device without the addition of DIO [92,93]. These results show that the addition of processing additives can be used as an alternative approach to replace thermal annealing for the fabrication of devices. On the other hand, residual additives were reported to give adverse effects on the photostability of solar cell devices [101]. Therefore, this approach is particularly beneficial for the fabrication of substrates of soft matters that is vulnerable to thermal stress; however, a careful approach will be required considering long-term stability.

Under mechanical deformation, most deformation of P3HT/PCBM blends is likely to arise in relatively softer amorphous parts. MO properties of interest are photocurrent and voltage that change under mechanical deformation. Optimized microstructure for MO behavior is not necessarily the same as one for solar cells. Each parameter and approach to control microstructures of P3HT/PCBM blends will need to be investigated for MO behavior.

### 2.5. Mechanical Properties

Mechanical properties of MO systems, for example, the tensile modulus and crack onset strain, are some of the essential factors for MO performance. Assuming independent optoelectronic properties on mechanical properties, smaller tensile modulus and greater crack onset strain of a MO system are anticipated to provide a broader sensing range and higher sensitivity. Measurement of mechanical properties of thin films is challenging because a thin film cannot be prepared as a free-standing specimen. Well-established methods for bulk specimens typically cannot work for thin films. Mechanical properties of conjugated polymer thin films have been examined indirectly using the buckling phenomenon of a thin film on an elastic supporting layer [19,102,103] or directly using a thin film floated on the water surface [29,104].

In the buckling method, a conjugated polymer thin film with around 100 nm thickness is transferred onto a pre-strained PDMS (typical pre-strain ~10%) with around 2 mm thickness [103]. When the pre-strain is released, buckling appears by the difference of moduli between a conjugated polymer thin film and a PDMS substrate. Based on a known tensile modulus of PDMS substrates (0.7 MPa), the tensile modulus of a conjugated polymer thin film can be estimated by measuring the bucking wavelength and thickness of a thin film. The crack onset strain is observed by a microscope overstrain. This method works well in the case of a hard thin film with a soft substrate. P3HT and PCBM thin films have two to three orders of magnitude greater tensile moduli than PDMS. In the method on the water surface, a conjugated polymer thin film is prepared on a water-soluble layer (e.g., PEDOT:PSS), and then water is allowed to penetrate into the water-soluble layer. Subsequently, the thin film is delaminated from the substrate and floated onto the water surface. Both ends of the floated thin film are gripped, and tensile tests carry out on the water surface. This method provides a direct measurement of the mechanical properties of conjugated polymer thin films. Dynamic tests are also available, for example, as a function of strain rate. However, this method has a limitation in temperature control. In both buckling and floating-on-water methods, the surface of a thin film is monitored by a microscope under tensile strain for the determination of the crack onset strain.

**Figure 10:**
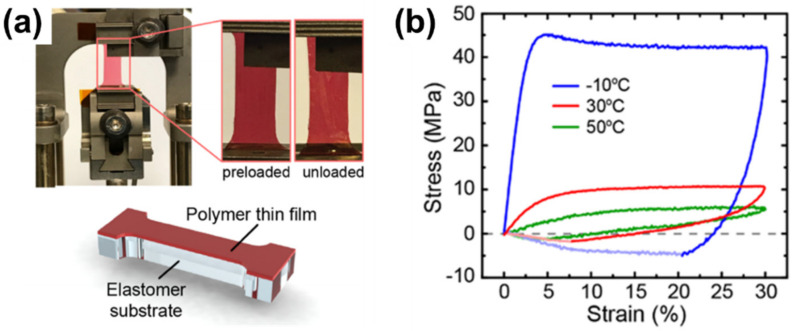
(**a**) A specimen of a P3HT thin film laminated on a thin PDMS substrate is loaded to a dynamic mechanical analyzer for tensile tests. (**b**) Stress–strain behavior of P3HT specimens at different temperatures: −10, 30, and 50 °C. Reprinted with permission from [105]. Copyright 2020 American Chemical Society.

Recently, a method using a conjugated thin film laminated on a thin elastomer was reported to address the limitation of the two methods [105]. As seen in Figure 10a, this approach employed a thin PDMS (~4 μm) as a supporting layer. A conjugated polymer thin film was transferred onto the thin PDMS layer, and then this composite specimen was examined by a dynamic mechanical analyzer in the tensile configuration. This approach could provide a broad measurement range, including strain and compression forces. Measurements at elevated temperatures were also available in this approach. The tensile modulus of a P3HT thin film exhibited a significant drop from 1.6 GPa at −10 °C to 0.2 GPa at 30 °C as heating above the glass transition temperature, as shown in Figure 10b. The tensile modulus increased as the strain rate increased, which is clear evidence of the viscoelastic behavior of a P3HT thin film [105].

For mechanical properties of P3HT thin films and P3HT/PCBM blend thin films, Lipomi et al. [102,106,107,108] have been exploring various alkyl side chains and different molecular weights. P3AT thin films with longer side chains exhibited smaller tensile moduli than that of a P3HT thin film. Thin films of poly(3-heptyl thiophene) (P3HpT) and poly(3-octyl thiophene) (P3OT) gave one-fifth tensile moduli than that of P3HT. In 50:50 P3AT:PCBM blend thin films, P3HpT and P3OT blends exhibited less than a half tensile moduli than that of a P3HT blend: 3.85, 1.79, and 0.51 GPa for blend thin films with P3HT, P3HpT, and P3OT, respectively. In photovoltaic devices with P3AT:PCBM blends, devices using P3HT and P3HpT outperformed a device using P3OT. Among three different P3ATs, P3HpT showed balanced performance in both mechanical properties and photovoltaic efficiency [106,107]. Therefore, a P3HpT thin film can be a promising alternative for a P3HT thin film for MO behavior. Cohesive and adhesive properties on P3AT thin films were examined by scratch tests using a stylus [108]. As the side-chain of P3AT is longer, cohesion and adhesion of a thin film decreased due to greater free volume and a lower glass transition temperature than those of a P3AT with a shorter side-chain. Cohesion and adhesion of P3AT thin films increased with molecular weights of P3AT. Molecular dynamics simulation revealed the normal forces at cohesive and adhesive failure were proportional to the degree of polymerization but not molecular weight. In other words, the greater strength of cohesion and adhesion was attributed to a long backbone of P3AT, allowing for the increased density of entanglements [108].

Tensile modulus and crack onset strain of P3HT/PCBM blend thin films are intimately correlated to the microstructure of thin films. P3HT/PCBM blend thin films containing various ordered P3HT portions were prepared by manipulating processing conditions. A fraction of the ordered P3HT in P3HT/PCBM thin films exhibited a direct correlation to photovoltaic performance and tensile moduli of thin films. The PCE increased from 1.44% to 3.67%, and tensile moduli increased from 1.74 to 2.02 GPa as the ordered fraction increased from 0.37 to 0.51. On the other hand, the crack onset strain was inversely proportional to a fraction of ordered P3HT in P3HT/PCBM blend thin films. A crack was observed at approximately 3% strain from a P3HT/PCBM thin film with the ordered P3HT fraction of 0.51, while a crack was not observed up to 80% strain from thin films with an ordered P3HT fraction of less than 0.40. The crack onset strain above 80% strain cannot be measured due to fracture of PDMS substrates [19].

The higher fraction of ordered P3HT in a P3HT/PCBM blend thin film is beneficial to the performance of a device. However, it gives only a few % of the crack onset strain, which is not sufficient for MO applications. To address this, an external approach can be considered. For example, an elastic polymer network can be incorporated in the P3HT/PCBM active layer to improve mechanical durability [109]. An elastic network was prepared using a P3HT/PCBM solution containing crosslinkable monomers, followed by casting a thin film and subsequent curing. The crack onset strain of P3HT/PCBM thin films increased from 6% to 28% by the incorporation of an elastic polymer network [109]. Furthermore, conjugated polymers can be employed as a substitute for PCBM in MO systems. Recent research efforts have been dedicated to polymer acceptors for excellent photovoltaic performance and potentially superior long-term stability [110,111,112]. Although improvement of photovoltaic performance does not necessarily bring excellent MO properties, polymer acceptor materials are a promising approach to enhance mechanical properties. Since PCBM is a crystalline single-molecule material, PCBM is more brittle than conjugated polymers and has two to three times greater tensile modulus than those of conjugated polymers [19,103,113]. Consequently, conjugated polymer/PCBM blend thin films give substantially lower toughness than a thin film substituting PCBM by another conjugated polymer acceptor [29]. MO systems using a conjugated polymer acceptor are a promising approach to enhance the mechanical toughness of conjugated polymer thin films.

In the previous sections, important parameters to be considered for MO systems were discussed. The majority of these parameters of conjugated polymer systems have been intensively studied for optoelectronic properties and photovoltaic performance. As optoelectronic properties do, MO properties are anticipated to interplay with all the aforementioned parameters of conjugated polymer systems. Therefore, comprehensive studies for these parameters are required in order to design a new conjugated polymer MO system.

## 3. Manufacturing of Conjugated Polymer MO Systems

Researchers have made an effort to scale up the fabrication of conjugated polymer thin films to broaden their applications in various fields. In solar cells, there is still a considerable gap between the photovoltaic performance of large-scale devices and that of lab-scale devices based on the spin coating process in an inert atmosphere [114,115,116,117]. The manufacturing of conjugated polymer MO devices is anticipated to face similar challenges. To address those, first, scalable processes need to be developed for the fabrication of conjugated polymer MO devices. Inkjet printing [24,115,116,117,118,119,120], spray coating [121], and roll-to-roll printing [18,114,122,123,124,125] are employed for the fabrication of large-scale conjugated polymer solar cells. Rigid parts must be substituted by flexible alternatives, including the active layer, electrodes, and substrates. Indium tin oxide (ITO) is widely used as a transparent electrode, but the brittle characteristic of ITO is a big challenge for scalable processes and flexible devices. Ryu et al. [24] fabricated strain sensing MO devices using a PEDOT:PSS layer as an electrode to substitute for ITO by the inkjet printing process. Strain sensors using ITO as an electrode worked in only 0.5% strain as sensing range, but sensing devices with a PEDOT:PSS electrode on PET substrate worked up to 2% strain. The transmittance of PEDOT:PSS layers was 90% to 57%, depending on thickness from 0.3 to 1.2 μm. The sheet resistance of PEDOT:PSS layers was 190 Ω/square for 0.3 μm thickness and decreased with thicker electrodes. Sheet resistance reached ~40 Ω/square for 0.6 μm thickness and maintained the level in thicker layers. Strain sensors using the PEDOT:PSS electrode exhibited a gage factor of 3.5 for 0.5% strain and 28 for greater than 1% strain [24]. The spray coating process is low-cost and can be combined with roll-to-roll fabrication. Spray-coating processes were employed for P3HT/PCBM photovoltaic devices using a PEDOT:PSS layer as an electrode. Highly conductive formulation of PEDOT:PSS solution was spray-coated on a glass substrate, and subsequently, P3HT:PCBM solution was spray-coated on the PEDOT:PSS layer for the fabrication of photovoltaic devices. The transmittance of a PEDOT:PSS electrode layer was 89% to 67%, depending on thickness from 100 to 350 nm. Corresponding sheet resistance was 358 to 63 Ω/square. Compared to ITO, thin PEDOT:PSS layers showed similar levels of transmittance, but PEDOT:PSS layers gave higher sheet resistance (~20 Ω/square of ITO). In photovoltaic performance, the short-circuit current *J*_SC_ decreased in a device with a PEDOT:PSS electrode due to its lower transmittance and higher series resistance, but the device gave a considerable efficiency (2.17% of PCE) compared to reference devices using an ITO electrode (2.86% of PCE) [121]. Device parameters of photovoltaic devices are listed in Table 1. ITO sputtered PET substrates were also employed for the fabrication of P3HT/PCBM solar cells by a roll-to-roll process. The photovoltaic modules having 120 cm^2^ of active area achieved 1.04% of PCE. Model devices using spin coating processes gave 2.7% PCE. in Table 1 [114]. This is an example of presenting challenges in scalable processes.

Next, processing conditions in scalable processes need to be investigated to reproduce lab-scale devices. A drying step is critical in the process to determine the final microstructure of the inkjet-printed active layer, which in turn affects the overall photovoltaic performance. If fluid mechanical phenomena associated with the drying process are understood, these phenomena, such as capillary flow, concentration gradient, and aggregation, can be utilized to improve the orientation and crystallinity of conjugated polymer thin films [126,127,128]. For inkjet printing, a processing solvent of fast evaporation is not favorable due to the clogging of the printing nozzle. Time-to-gelation in inkjet printing was controlled by processing parameters, for example, processing solvent, the temperature of a substrate, and chemical properties of materials, etc. A mixture of o-dichlorobenzene (oDCB) and 1,3,5-trimethyl benzene (mesitylene) was employed as a processing solvent in inkjet printing of the P3HT/PCBM active layer onto PEDOT:PSS/ITO substrates. As presented in Table 1, photovoltaic devices achieved 3.47% PCE by varying regioregularity of P3HT and inkjet printing parameters, such as drop velocity, droplet volume, and drop spacing [115]. Another solvent mixture of chlorobenzene and trichlorobenzene was employed for inkjet-printed P3HT/PCBM active layers. Photovoltaic devices made by inkjet printing gave comparable performance (2.40% PCE) to a reference device made by spin coating (2.64% PCE). However, when both a PEDOT:PSS layer and the active layer were inkjet-printed, photovoltaic performance declined significantly (1.54% PCE) [117]. Scalable processes and their process conditions need to be optimized not only for the active layer but also for other components of devices.

Lastly, long-term stability is an essential factor for large-scale devices. Particularly for MO systems, important requirements are mechanical resilience against fatigue and thermal stability. Thermal stability of MO properties is determined by the stability of photocurrent under temperature changes, and thus the stability of microstructure at operating temperature. Glass transition temperature of conjugated polymers is a dictating factor for the stability of microstructure and viscoelastic behavior of a MO thin film. Very recently, the glass transition of conjugated polymers, including P3ATs, has been characterized by rational prediction based on sorting elemental chemical structure by the level of rigidity and flexibility [129,130]. Glass transition temperature of a MO thin film needs to be controlled depending on a targeted operating temperature. The mechanical resilience of solar cell devices has been examined under bending and torsion cycles. Photovoltaic modules were printed by a roll-to-roll process in a dimension of 100 mm × 142 mm. In photovoltaic modules integrated with PEDOT:PSS layer showed failure after 5000–8000 cycles. When the PEDOT:PSS layer was substituted by silver nanowires, no failure was observed for up to 15,000 cycles. Under bending cycles, decohesion and subsequent deadhesion were observed between the PEDOT:PSS layer and the P3HT/PCBM active layer, which resulted in delamination of the module. The torsion that exerted stronger stress caused the failure at fewer cycles. As consistent with the bending tests, photovoltaic modules with silver nanowires exhibited improved mechanical resilience than those with a PEDOT:PSS layer. Under torsion tests, crazing appeared first and then cracks propagated from the edge of modules. In the study, however, impacts on mechanical resilience by different electrode materials were not conclusively investigated because two devices were prepared on PET substrates in different thicknesses: 160 μm for a PEDOT:PSS device and 190 μm for a silver nanowire device. A PET substrate was the most durable layer in the device stack as an encapsulation barrier. The improved mechanical resilience from the silver nanowire device could be partially due to the thicker PET substrate [18]. The stability of P3HT/PCBM photovoltaic devices was examined under oxygen and humidity. Normal architecture and inverted architecture showed similar PCE (~2.7%) in model devices by spin coating. Normal devices using aluminum electrodes lost photovoltaic performance in less than 50 h under a humid atmosphere, while inverted devices using silver electrodes were stable for 200 h. Under oxygen, in contrast, normal devices retained 70% of the initial performance, while inverted devices lost the majority of photovoltaic performance within 10 h. Under ambient atmosphere having both oxygen and humidity, neither normal nor inverted device was stable [114]. As described above, materials of each layer and device architecture need to be investigated with respect to the stability of device performance. Sealing methods and materials of an encapsulation layer are also important factors for mechanical resilience and long-term stability of MO devices.

## 4. Summary and Outlook

The mechano-optoelectronic (MO) properties of P3HT/PCBM blend thin films are attributed to changes in the microstructure under mechanical deformation, which leads to the corresponding changes in optoelectronic properties. Since semicrystalline P3HT has near or less than 50% crystallinity in P3HT/PCBM blend thin films, the microstructure of P3HT/PCBM thin films is composed of ordered P3HT regions embedded in a mixture of amorphous P3HT and PCBM. Under tensile strain, most elongation occurs at soft amorphous regions, and hard ordered regions rotate to be aligned with the direction of elongation. Therefore, the extent of the changes will be correlated to the degree of crystallinity, size of crystallites, and the initial alignment/orientation of crystallites before mechanical deformation is applied. Those factors can be manipulated by the design of materials, for example, regioregularity, molecular weight, and length of alkyl side chains. Processing conditions, including the selection of processing solvents, the composition of P3HT/PCBM blends, and post-treatments, are expected to help the design of MO systems for enhanced sensitivity and sensing range. Moreover, polymeric acceptor materials are a promising approach to improve the mechanical toughness of MO systems.

Manufacturing of MO conjugated polymer thin films can utilize inkjet printing, spray coating, and roll-to-roll printing, which have been used for the fabrication of photovoltaic devices. All components of devices need to be substituted with flexible parts, including electrodes and substrates as an encapsulation barrier. Reproducible performance of lab-scale devices and mechanical resilience is also essential to realize these scalable processes. For applications, MO conjugated polymer thin films are expected to serve as a sensing component in structural health monitoring, biomedical devices, and wearable devices. Furthermore, one of the most significant advantages of thin-film devices is the ability to stack up multiple layers. A multilayer thin film MO device was prepared by a combination of a P3HT/PCBM blend thin film and a polyaniline thin film. The multilayer device presented the capability of multimodal detection for changes in tensile strain and pH simultaneously [23]. In the future, these MO conjugated polymer films are expected to provide multiple functionalities in tandem with an additional sensing layer or a light-emitting layer.

## Figures and Tables

**Table 1:** Device parameters of P3HT/PCBM solar cells by scalable processes.

	*J*_SC_ (mA/cm^2^)	*V*_OC_ (V)	*FF*	PCE (%)	Ref.
Normal architecture by roll-to-roll printing	9.30	0.56	0.52	2.70	[114]
Inverted architecture by roll-to-roll printing	8.85	0.54	0.56	2.67	[114]
Inkjet printing on ITO substrate	10.05	0.54	0.64	3.47	[115]
Spin coating as reference	9.69	0.60	0.45	2.64	[117]
P3HT/PCBM by inkjet printing	9.34	0.57	0.45	2.40	[117]
P3HT/PCBM and PEDOT:PSS by inkjet printing	8.94	0.51	0.34	1.54	[117]
Spray-coated P3HT/PCBM on ITO electrode	8.06	0.59	0.60	2.86	[121]
Fully spray-coated device with P3HT/PCBM on PEDOT:PSS electrode	6.62	0.61	0.54	2.17	[121]

## Data Availability

Data sharing not applicable.
